# Associations between general practice characteristics and chest X-ray rate: an observational study

**DOI:** 10.3399/BJGP.2021.0232

**Published:** 2021-12-14

**Authors:** Stephen H Bradley, Matthew Barclay, Benjamin Cornwell, Gary A Abel, Matthew EJ Callister, Mayam Gomez-Cano, Thomas Round, Bethany Shinkins, Richard D Neal

**Affiliations:** Leeds Institute of Health Sciences, University of Leeds, Leeds.; Epidemiology of Cancer Healthcare Outcomes Group, University College London, London.; Emergency Medicine, Leeds Teaching Hospitals NHS Trust, Leeds.; College of Medicine and Health, University of Exeter, Exeter.; The Leeds Centre for Respiratory Medicine, Leeds Teaching Hospitals NHS Trust, Leeds.; College of Medicine and Health, University of Exeter, Exeter.; School of Population Health and Environmental Sciences, King’s College London; National Cancer Registration and Analysis Service, Public Health England, London.; Test Evaluation Group, Leeds Institute of Health Sciences, University of Leeds, Leeds.; Leeds Institute of Health Sciences, University of Leeds, Leeds.

**Keywords:** cancer diagnosis, chest X-ray, general practice, lung cancer, outcome assessment, health care, referral and consultation

## Abstract

**Background:**

Chest X-ray (CXR) is the first-line test for lung cancer in many settings. Previous research has suggested that higher utilisation of CXR is associated with improved outcomes.

**Aim:**

To explore the associations between characteristics of general practices and frequency of investigation with CXR.

**Design and setting:**

Retrospective observational study of English general practices.

**Method:**

A database was constructed of English general practices containing number of CXRs requested and data on practices for 2018, including patient and staff demographics, smoking prevalence, deprivation, and patient satisfaction indicators. Mixed-effects Poisson modelling was used to account for variation because of chance and to estimate the amount of remaining variation that could be attributed to practice and population characteristics.

**Results:**

There was substantial variation in GP CXR rates (median 34 per 1000 patients, interquartile range 26–43). Only 18% of between-practice variance in CXR rate was accounted for by recorded characteristics. Higher practice scores for continuity and communication skills, and higher proportions of smokers, Asian and mixed ethnic groups, and patients aged >65 years were associated with increased CXR rates. Higher patient satisfaction scores for access and greater proportions of male patients and patients of Black ethnicity were associated with lower CXR rates.

**Conclusion:**

Substantial variation was found in CXR rates beyond that expected by chance, which could not be accounted for by practices’ recorded characteristics. As other research has indicated that increasing CXR rates can lead to earlier detection, supporting practices that currently investigate infrequently could be an effective strategy to improve lung cancer outcomes.

## INTRODUCTION

GPs play a crucial role in the timely diagnosis of lung cancer.^1^ Most cancer patients are seen by a GP before diagnosis^2^^–^^4^ and, in England, almost half of all lung cancer diagnoses result from GP referrals.^5^ Chest X-ray (CXR) is widely used by GPs as a first-line test for suspected lung cancer.^6^^,^^7^ In the UK, clinical guidelines recommend that GPs investigate lung cancer symptoms using CXR, with the exception of unexplained haemoptysis, which qualifies for immediate referral without suspicious findings on CXR.^8^ Lung cancer outcomes in the UK lag behind those of similar countries.^9^ Increasing the proportion of symptomatic patients who receive investigation is likely to be vital to improving diagnosis at earlier stages and, consequently, survival.^10^

Previous research has demonstrated that patients with lung cancer are more likely to be diagnosed with earlier-stage disease and have improved survival if they are registered at general practices with higher rates of urgent referral for suspected cancer.^11^^,^^12^ Further evidence from a previous symptom awareness campaign suggests that increasing CXR rates may contribute to diagnoses at earlier stages of disease and improved survival.^13^ Understanding the underlying reasons for variation in CXR rate could help to develop interventions aimed at increasing rates in practices with lower rates.

To facilitate comparative evaluation, primary care performance in cancer diagnosis, activity indicators have been compiled for all general practices in England.^14^ These include frequency of urgent referrals for suspected cancer, which are presented alongside data on demography and practice disease registers. Analysis of these data has demonstrated substantial variation in these activity indicators between general practices, beyond that which can be accounted for by chance variation, even after adjusting for differing practice populations.^15^ Despite CXR being a widely used and accessible test,^16^ CXR rate across general practices and factors that drive variation between practices remains relatively unexplored. This study explores whether population and practice characteristics are associated with frequency of CXR investigation in general practices across England. The purpose, in exploring variation in utilisation in CXR, is primarily because of the importance of this modality for lung cancer detection, although the study reports total number of CXRs undertaken, not just those that were requested because of suspected lung cancer.

**Table:** How this fits in

Abnormal findings on chest X-rays that have been requested by GPs because of symptoms are an important route to lung cancer diagnosis. Previous research has suggested that increased rates of chest X-ray and urgent referral for suspected cancer may be associated with earlier stage at diagnosis for lung cancer. This study demonstrates that there is substantial variation in rates of investigation between practices, and that only a small proportion of that variation is owing to examined population and practice characteristics. Encouraging practices that have low chest X-ray rates to lower their thresholds for investigation could prove to be an effective strategy to detect lung cancer earlier and improve outcomes.

## METHOD

Data were obtained for all English general practices with list sizes over 1000 patients and for which data were available on numbers of patients who were investigated by general practices with CXR. Using methods similar to those employed in a previous study on variation in investigation with gastrointestinal endoscopy, associations were examined between CXR use and characteristics of the practices and their populations.^17^

### Data

The number of patients registered at each practice who had at least one CXR in 2018 requested by their GP was obtained from the Diagnostic Imaging Dataset.^18^ Data on general practices and their populations were obtained from Public Health England’s general practice profiles, the General Practice Patient Survey (https://gp-patient.co.uk), and NHS General and Personal Medical Services datasets.^14^^,^^19^ All data pertained to 2018, except for the Index of Multiple Deprivation (IMD) and ethnicity, which are not reported directly for practice populations but are aggregated estimates based on the 2011 national census and IMD 2015. The formulation of IMD measures and ethnicity estimates for practice populations has been described previously.^20^^,^^21^

Data on six sets of variables relating to practice populations and a further eight relating to the general practices themselves were obtained, described in full in the preregistered analysis plan.^22^

Practice scores from the general practice survey for 2018 were included in the analysis, adjusted for age, long-term conditions, ethnicity, and deprivation, as described elsewhere.^23^ The population and practice characteristics were selected based on those included in previous studies where the performance of general practices in England have been compared using similar datasets.^17^^,^^21^^,^^23^ A decision was taken to include data on the numbers of patients who were on heart failure and chronic obstructive pulmonary disease (COPD) registries as these are common conditions that present with symptoms that may be investigated with CXR.^22^

### Analysis

A mixed-effects Poisson regression model was used, including a random effect for general practice, to determine the extent to which variation in numbers of patients who had a CXR between practices could be attributable to population and/or practice characteristics. Mixed-effects models can estimate the overall underlying variation between practices after removing the role of chance because of small numbers.^24^ To account for differing practice sizes, an offset variable was included that was the log of the practice list size.

Three further iterations of the model were run, including: 1) the variables relating to the practice population characteristics; 2) the variables relating to practice characteristics; and 3) both groups of variables combined. The percentage of the variation in frequency of CXR investigation that each model could account for was estimated. The median incidence rate ratio (MIRR)^25^ was also determined as an alternative means of expressing the degree of variation that was accounted for by each version of the model. MIRR is a statistic that measures the median relative change in a rate when two identical subjects (that is, practices) from randomly selected clusters ordered by rate are compared.

As continuous exposure variables have different distributions across practices, and to facilitate comparisons of their effect sizes, values were standardised by subtracting the mean value across all practices from actual value then dividing by 1.35 standard deviations. One unit difference in these standardised scores corresponds to a change between the 25th and 75th centile of normally distributed continuous variables. The resulting rate ratios correspond to the change resulting from moving from the 25th to the 75th centile of the exposure variable (practice team or practice population characteristic) of interest.

Given the large sample size and the multiple variables being studied, statistically significant associations with little clinical importance were anticipated. Results were therefore presented with ‘cut-offs’ for rate ratios; a difference of ≥4% (that is, ≤0.96 or ≥1.04) and with *P*<0.01. The pre-registered analysis plan provides further details on study data and analyses.^24^

## RESULTS

**Figure 1.**
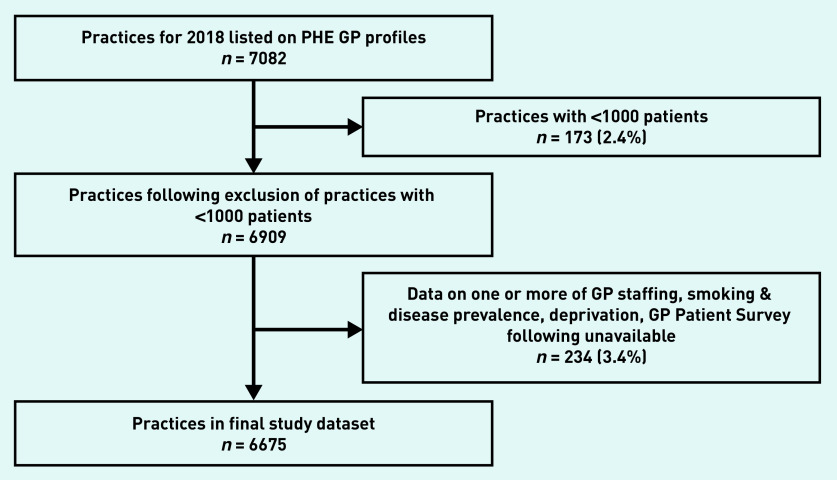
*Number of practices that were excluded. More information can be found on excluded practices in Supplementary Table S1. PHE = Public Health England.*

Following exclusion of practices with <1000 patients (*n* = 173), data for 6909 practices remained. A further 234 practices (3.4%) were excluded because data were not available (Figure 1 and Supplementary Table S1). The characteristics of the 6675 practices included in the analysis are outlined in Table 1. A median of 33.8 CXRs were performed per 1000 patients, with substantial variation (interquartile range [IQR] 25.5–42.6) between practices.

**Table 1.** Practice-level variables and practice characteristics used in analysis. All data that does not pertain to 2018 is indicated by a footnote

**Variables and characteristics**	**Median (IQR)**	**10th–90th centiles**	**Median (IQR) for general practices within 0–10th centiles of CXR rate (<16.5 per 1000 patients) (*n*= 667)**	**Median (IQR) for general practices within 90–100th centiles of CXR rate > (51.2 per 1000 patients) (*n*= 668)**
CXRs per 1000 patients	33.8 (25.5–42.6)	16.5–51.2	—	—

Total CXRs per practice^a^	250 (135–395)	70–560	35 (5–85)	368 (255–570)

**Practice population characteristics**				

Percentage of patients who are male	49.7 (48.9–50.8)	48.2–52.7	50.2 (49.1–52.2)	49.5 (48.6–50.1)

Percentage of patients aged ≥65 years	17.6 (12.4–21.9)	8.2–26.0	11.4 (6.7–18.7)	21.1 (17.3–25.4)

Percentage of patients who are smokers	16.5 (12.9–20.6)	10.4–24.1	16.8 (13.2–20.5)	17.0 (13.5–21.1)

Percentage of patients on practice chronic obstructive pulmonary disease register	1.9 (1.3–2.5)	0.9–3.2	1.4 (0.7–2.1)	2.7 (2.2–3.3)

Percentage of patients on heart failure register	0.9 (0.6–1.7)	0.4–1.5	0.6 (0.4–0.9)	1.8 (0.9–1.5)

Ethnicity category estimates, %^b^				
White	92.4 (75.3–97.3)	50.6–98.2	80.8 (58.6–96.3)	96.8 (92.1–98.1)
Mixed/multiple ethnic groups	1.7 (1.0–3.5)	0.7–5.2	3.2 (1.0–4.9)	1.1 (0.8–1.7)
Asian/Asian British	3.6 (1.2–11.0)	0.7–25.6	8.1 (1.9–15.5)	1.4 (0.8–3.8)
Black/African/Caribbean/Black British	1.0 (0.3–4.9)	0.2–12.0	3.7 (0.5–11.4)	0.4 (0.2–1.3)
Other ethnic groups	0.4 (0.2–1.5)	0.1–3.5	1.4 (0.2–3.1)	0.2 (0.1–0.5)

**General practice characteristics**				

Patients per full-time equivalent GP, *n*	1881 (1440–2459)	1157–3404	2110 (1630–2829)	1629 (1290–2196)

Percentage GPs who are male	51.1 (36.2–68.7)	21.3–97.3	53.0 (36.8–75.8)	53.4 (38.1–70.4)

Percentage GPs who are UK qualified	75.0 (50.0–100.0)	0–100	69.6 (33.3–92.9)	75.0 (50.0–100.0)

GP age, years, mean	46 (43–50)	40–56	47 (43–53)	45 (42–50)

Practice list size, *n*	7622 (4869–11 141)	3258–14 782	6829 (4486–11 008)	6345 (4274–9595)

Percentage who gave highest rating for general practice survey for:				
Helpfulness of reception staff	48.5 (39.7–58.2)	32.7–67.1	49.7 (41.2–58.8)	51.5 (42.1–62.1)
Ability to see preferred GP (continuity)	54.7 (48.8–60.8)	43.5–66.5	54.3 (47.8–60.4)	56.9 (51.2–63.1)
Ability to book appointment (access)	25.0 (17.4–34.7)	13.0–46.4	27.9 (20.1–38.0)	25.1 (16.6–36.5)
Healthcare professional communication skills	78.4 (72.3–84.0)	66.1–88.3	78.4 (71.4–83.9)	79.6 (73.6–85.6)

**Categorical variables**	***n* (%)^c^**	—	***n* (%)^c^**	***n* (%)^c^**

Deprivation fifth^d^				
F1	1360 (20.4)	—	75 (11.2)	83 (12.4)
F2	1357 (20.3)		106 (15.9)	148 (22.2)
F3	1337 (20.0)	—	151 (22.6)	133 (19.9)
F4	1444 (21.6)	—	226 (33.9)	154 (23.1)
F5	1177 (17.6)	—	109 (16.3)	150 (22.5)

Single-handed status				
Yes	410 (6.1)	—	60 (9.0)	39 (5.8)
No	6265 (93.9)	—	607 (91.0)	629 (94.2)

Practice location				
Urban	5695 (85.3)	—	551 (82.6)	569 (85.2)
Rural	980 (14.7)	—	116 (17.4)	99 (14.8)

Practice involved in postgraduate GP training				
Yes	2486 (37.2)	—	184 (27.6)	277 (41.5)
No	4189 (62.8)	—	483 (72.4)	391 (58.5)

a *In order to maintain patient anonymity, Diagnostic Imaging Dataset rounds CXR counts for each practice to nearest 5 and counts of* <*3 are suppressed. In this study ‘2’ was substituted for practices with counts of* <*3.*

b *The ethnic composition of practice populations estimated by applying 2011 census data to the 2015 practice populations. These estimates were obtained from Public Health England.*

c *As a result of rounding, not all percentages add precisely to 100.*

d *Derived from Index of Multiple Deprivation practice scores for 2015. F1 = least deprived; F5 = most deprived. CXR = chest X-ray. IQR = interquartile range.*

Less than a fifth of this variation was accounted for by combined population and practice characteristics (Table 2). Of the two, population characteristics were found to be more important, resulting in a 16.4% reduction in between-practice variance compared with only 2.8% for practice characteristics.

**Table 2.** Extent of between-practice variation explained by each model expressed as percentage reduction in random-effects variance and by median incidence rate ratio

**Model**	**Random-effects variance^a^**	**Percentage reduction in variance**	**MIRR^b^**	**Ratio of MIRR to that of null model**
Null (random effect only)	0.58	—	2.07	—
Population characteristics only	0.50	16.4	1.95	0.95
Practice characteristics only	0.56	2.8	2.05	0.99
Both population and practice characteristics	0.49	17.9	1.95	0.94

a *This is the variance between practices measured on the log-scale.*

b *This is the ratio of the CXR rate for a practice at the 75 th centile of the CXR utilisation against the CXR rate for a practice at the 25 th centile of CXR utilisation, estimated using the random-effects variance. CXR = chest X-ray. MIRR = median incidence rate ratio.*

A hypothetical example of how changes in population and practice characteristics could be expected to affect numbers of CXRs performed is presented in Table 3. The MIRR was 1.95 for the model that included both sets of characteristics, and 1.95 and 2.05 for models with only population and practice characteristics, respectively (Table 2). Adjusted and unadjusted associations between CXR rate and population and practice characteristics are presented in Table 4 and Supplementary Table S2.

**Table 3.** Theoretical example of how changes in population and practice characteristics determined from the adjusted model would be expected to affect the number of patients receiving CXR in a year in a practice with 8000 patients, based on the mean CXR rate of 34 CXRs per 1000 patients^a^

**Characteristic**	**Distribution of characteristics**			**CXRs, *n* (95% CI)**		**Difference in number of CXRs between 75th and 25th centile, *n* (95% CI)**

	**Overall, mean (SD)**	**25th centile**	**75th centile**	**25th centile**	**75th centile**	
**Practice population characteristics**						

Percentage of patients who are male	50.1 (2.4)	48.6	51.7	277 (273 to 281)	265 (252 to 269)	−8 (−4 to −19)

Percentage of patients aged ≥65 years	17.4 (6.8)	12.8	22.0	226 (219 to 233)	316 (309 to 323)	97 (76 to 104)

Percentage of patients who are smokers	17.1 (5.7)	13.3	20.9	247 (241 to 253)	295 (289 to 301)	54 (35 to 61)

Percentage patients on heart failure registry	0.9 (0.4)	0.6	1.2	260 (253 to 267)	282 (278 to 287)	28 (14 to 32)

Ethnicity category, %						
Mixed/multiple ethnic groups	2.4 (1.8)	1.2	3.6	260 (253 to 267)	282 (275 to 289)	29 (7 to 36)
Asian/Asian British	9.3 (13.4)	0.2	18.0	248 (243 to 253)	294 (289 to 299)	51 (35 to 56)
Black/African/Caribbean/Black British	4.1 (6.7)	0.00	8.6	280 (275 to 286)	262 (256 to 268)	−13 (−7 to −30)

**General practice variables**						

Percentage who gave highest rating for general practice survey for:						
Helpfulness of reception staff	49.4 (13.1)	40.5	58.2	279 (274 to 285)	263 (257 to 268)	−11 (−6 to −27)
Ability to book appointment (access)	27.6 (13.3)	18.6	36.6	281 (276 to 285)	263 (257 to 268)	−15 (−10 to −28)
Ability to see preferred GP (continuity)	54.9 (8.8)	48.9	60.8	261 (256 to 266)	281 (276 to 286)	25 (9 to 30)
Healthcare professional communication skills	77.7 (8.6)	71.9	83.5	264 (259 to 269)	278 (273 to 283)	19 (4 to 24)

**Categorical variables**		**Difference in number of CXRs expected to result from theoretical change in deprivation from deprivation fifth 1, *n* (95% CI)**				

Deprivation fifth 3			35 (19 to 50)			

Deprivation fifth 4			44 (26 to 61)			

a *In the cases of variables for which moving from 25 th to 75 th centile would result in fewer CXRs, the example assumes that the variables follow a normal distribution. Only variables with effect sizes ≥1.04 or ≤0.96 (*P<*0.01) are included. CXR = chest X-ray. SD = standard deviation.*

**Table 4.** Adjusted associations between CXR rates with population and general practice characteristics in English general practices in 2018^a^

**Characteristic**	**Rate ratios^b^ (95% CI)**	***P*-value**
**Practice population characteristics**		

Percentage of patients who are male	**0.96 (0.93 to 0.99)**	**0.004**

Percentage of patients aged ≥65 years	**1.36 (1.30 to 1.42)**	**<0.001**

Percentage of patients who are smokers	**1.18 (1.13 to 1.24)**	**<0.001**

Percentage of patients on practice chronic obstructive pulmonary disease register	1.05 (1.01 to 1.10)	0.018

Percentage of patients on heart failure register	**1.09 (1.05 to 1.12)**	**<0.001**

Ethnicity category, %		
Mixed/multiple ethnic groups	**1.08 (1.03 to 1.14)**	**0.003**
Asian/Asian British	**1.17 (1.13 to 1.22)**	**<0.001**
Black/African/Caribbean/Black British	**0.93 (0.89 to 0.97)**	**0.002**
Other ethnic groups	0.97 (0.94 to 1.01)	0.099

Deprivation fifth^c^		
F2	0.94 (0.88 to 0.99)	0.019
F3	**0.87 (0.82 to 0.93)**	**<0.001**
F4	**0.84 (0.77 to 0.90)**	**<0.001**
F5	0.93 (0.84 to 1.03)	0.161

**General practice characteristics**		

Patients per full-time equivalent GP	1.00 (0.98 to 1.03)	0.729

Percentage GPs who are male	0.97 (0.95 to 0.99)	0.017

Percentage GPs who are UK qualified	1.00 (0.97 to 1.03)	0.853

GP age, mean	0.99 (0.96 to 1.02)	0.590

Practice list size	**0.95 (0.92 to 0.97)**	**<0.001**

Single-handed practice	0.99 (0.91 to 1.07)	0.814

Practice involved in postgraduate GP training	1.02 (0.98 to 1.07)	0.369

Rural location	1.03 (0.99 to 1.09)	0.165

Percentage who gave highest rating for general practice survey for:		
Helpfulness of reception staff (access)	**0.94 (0.90 to 0.98)**	**0.002**
Ability to book appointment (access)	**0.93 (0.90 to 0.96)**	**<0.001**
Ability to see preferred GP (continuity)	**1.07 (1.04 to 1.11)**	**<0.001**
Healthcare professional communication skills	**1.05 (1.01 to 1.09)**	**0.007**

a *The rate or odds ratios correspond to the change in the rate resulting from moving from the 25 th to the 75 th centile of the exposure variable (practice team or practice population characteristic) of interest. Bold fonts used for rate ratio values ≥1.04 or ≤0.96 where* P <*0.01.*

b *For categorical values these are odds ratios.*

c *Derived from Index of Multiple Deprivation practice scores for 2015. F1 = least deprived; F5 = most deprived. CXR = chest X-ray.*

A small proportion of practices were found to have undertaken fewer than three CXRs (*n* = 127, 1.9%). The characteristics of these practices are described in Supplementary Table S3. As it was judged that such low rates of investigation with CXR could represent an error of reporting, a post hoc sensitivity analysis was undertaken on associations between population and practice characteristics, excluding these practices. The sensitivity analysis (Table 5) provided broadly consistent findings with the main analysis, with differences noted below. Standardised variables for all 6675 practices are included in Supplementary Dataset S1.

**Table 5.** Sensitivity analysis of adjusted associations between CXR rates with population and general practice characteristics for 2018, excluding practices that performed <3 CXRs in 2018 (*n* = 127, 1.9%)^a^

**Characteristic**	**Rate ratios^b^ (95% CI)**	***P*-value**
**Practice population characteristics**		

Percentage patients male	**0.96 (0.94 to 0.98)**	**<0.001**

Percentage of patients aged ≥65 years	**1.26 (1.21 to 1.30)**	**<0.001**

Percentage of patients who are smokers	**1.11 (1.07 to 1.14)**	**<0.001**

Percentage of patients on chronic obstructive pulmonary disease register	**1.16 (1.12 to 1.20)**	**<0.001**

Percentage of patients on heart failure register	**1.07 (1.04 to 1.09)**	**<0.001**

Ethnicity category, %		
Mixed/multiple ethnic groups	0.99 (0.95 to 1.03)	0.62
Asian/Asian British	**1.14 (1.11 to 1.17)**	**<0.001**
Black/African/Caribbean/Black British	0.98 (0.95 to 1.02)	0.311
Other ethnic groups	0.98 (0.96 to 1.01)	0.236

Deprivation fifth^c^		
F2	0.95 (0.91 to 0.99)	0.020
F3	**0.92 (0.88 to 0.97)**	**0.001**
F4	**0.91 (0.86 to 0.97)**	**0.002**
F5	0.93 (0.87 to 1.00)	0.054

**General practice characteristics**		

Patients per full-time equivalent GP	1.00 (0.99 to 1.02)	0.635

Percentage of GPs who are male	0.98 (0.97 to 1.00)	0.093

Percentage of GPs who are UK qualified	0.99 (0.99 to 1.01)	0.251

GP age, mean	0.99 (0.97 to 1.01)	0.318

Practice list size	**0.93 (0.90 to 0.94)**	**<0.001**

Single-handed practice	0.97 (0.92 to 1.03)	0.381

Practice involved in postgraduate GP training	**1.06 (1.02 to 1.09)**	**0.001**

Rural location	1.03 (0.99 to 1.06)	0.137

Percentage who gave highest rating for general practice survey for:		
Helpfulness of reception staff (access)	**0.95 (0.92 to 0.98)**	**0.001**
Ability to book appointment (access)	**0.95 (0.93 to 0.98)**	**<0.001**
Ability to see preferred GP (continuity)	**1.06 (1.03 to 1.09)**	**<0.001**
Healthcare professional communication skills	1.03 (1.00 to 1.06)	0.052

a *The characteristics of these practices are outlined in Supplementary Table S3. The rate or odds ratios correspond to the change in the rate resulting from moving from the 25th to the 75th centile of the exposure variable (practice team or practice population characteristic) of interest. Bold fonts used for rate ratio values ≥1.04 or ≤0.96 where* P <*0.01.*

b *For categorical values these are odds ratios.*

c *Derived from Index of Multiple Deprivation practice scores for 2015. F1 = least deprived; F5 = most deprived. CXR = chest X-ray.*

### Population characteristics

Practices with higher proportions of smokers, patients on heart failure registers, and those aged ≥65 years had higher rates of investigation with CXRs (Table 4). On excluding practices that performed <3 CXRs, an association between higher CXR rates and proportions of patients on COPD registers was demonstrated (Table 5). Practices with higher estimated proportions of patients belonging to mixed/multiple ethnic groups or Asian/Asian British ethnic categories also had higher CXR rates. CXR rates were lower in practices with higher proportions of male patients and estimated proportions of patients in the Black/African/Caribbean/Black British ethnic category. When practices that performed <3 CXRs were excluded, the associations between mixed/multiple ethnic groups and increased CXR rates and the Black/African/Caribbean/Black British ethnic category with reduced CXR rates were not demonstrated (Table 5).

There was no consistent relationship with deprivation but some suggestion that more deprived groups had lower adjusted rates of investigation, with odds ratios for deprivation of 0.84 (95% confidence interval (CI) = 0.77 to 0.90, *P*<0.001) for deprivation fifth four versus one, and 0.93 (95% CI = 0.84 to 1.03, *P*<0.161) for deprivation fifth five versus one. An exploratory, post hoc analysis including the IMD score as a linear continuous variable found no evidence of a relationship (*P* = 0.7, data not shown).

### Practice characteristics

Practices with larger list sizes had lower rates of CXR, although higher numbers of patients per full-time equivalent was not shown to be associated with lower rates of CXR. General practice location, GP age, single-handed status, and involvement in GP training were not associated with differences in CXR rate. On excluding practices that performed <3 CXRs, an association between involvement in GP training was associated with increased CXR rates. Practices that achieved the highest scores for general practice survey items pertaining to access (helpfulness of receptionist and ability to book appointment) had reduced CXR rates while items pertaining to continuity (ability to see preferred GP) and healthcare professional communication skills were associated with higher practice CXR rates. The association between healthcare professional communication skills and increased CXR rates did not meet predefined significance thresholds when practices that had <3 CXRs were excluded (Table 5).

## DISCUSSION

### Summary

To the authors’ knowledge, this is the most comprehensive investigation published on the population and practice characteristics associated with rates of CXR investigation by GPs. The resulting insights are primarily of interest because of the role of CXR in lung cancer detection from primary care, although the study included counts of all CXRs, regardless of indication. Several population and practice characteristics were found to be associated with differences in CXR rates, but the effect size of most of these was small. The characteristic with the largest effect size was the proportion of patients aged ≥65 years. Characteristics relating to practice populations were found to have a much greater association with differences in CXR rates than characteristics of the practices themselves.

As well as age, the most important population characteristics associated with higher CXR rates were smoking and heart failure prevalence, and higher estimated proportions of patients from Asian and mixed ethnicity groups. Lower CXR rates were associated with practices with higher proportions of Black patients and male patients. However, in combination, all population characteristics could only account for around a sixth of observed betweenpractice variation in investigation with CXR. Characteristics of the practices themselves (for example, staffing, training status, and location) accounted for even less of this variation and few of these individual-practice characteristics were linked to appreciable differences in CXR rates. Achievement of the highest scores in GP survey items relating to access was associated with lower CXR rates while achievement of highest scores for items relating to continuity of care and communication skills were associated with higher CXR rates.

### Strengths and limitations

This study used a large national sample of general practices with analysis performed according to a pre-registered plan. In studies of this type, with a large sample size and numerous covariates, there is a risk that statistically significant differences are observed that have no or negligible importance. In the present study, the use of pre-specified cut-off values provides some confidence that the observed associations reflect meaningful differences in investigation rates.^26^

Suspected lung cancer is only one possible indication for investigation with CXR from primary care. When GPs suspect other illnesses they may also arrange CXRs as several of these pathologies may cause similar symptoms such as cough and shortness of breath, and in many instances one rationale for organising CXR may be to exclude malignant disease as well as to confirm a primary differential such as heart failure or pneumonia. Previous audit evidence suggests that investigation with CXR because of symptoms, even when lung cancer is not explicitly suspected, is an important route to diagnosis.^27^ In this study it was not possible to capture the indication for CXR and it is important to acknowledge that the proportion of CXRs that were arranged for suspected lung cancer is unknown.

A small proportion of practices (3.4%) were excluded from the analysis because data for the practice were not available for ≥1 of the sets of variables that were studied (see Supplementary Table S1). Excluded practices had a similar rate of CXR; however, these practices had fewer registered patients (median of 4855 versus 7622), higher rates of smoking (median 20.3% versus 16.5%), were more frequently located in the most deprived fifth (32.0% versus 17.6%), and were more often single-handed practices (37.2% versus 6.1%). A proportion of practices (1.9%) were recorded as having performed <3 CXRs. It is possible that the number of CXRs is recorded incorrectly in the Diagnostic Imaging Dataset for these practices, therefore associations are also reported excluding these practices (Table 5).

### Comparison with existing literature

A study by O’Dowd *et al* determined age- and sex-standardised CXR rates for 71 general practices in England and reported a similar median rate (four CXRs per 100 patients per year) to the present study but an even wider variation in CXR rates (IQR 3–6).^28^ Another study based in a single city has also demonstrated wide variation in CXR rates.^29^ The present study draws from a much larger sample of practices (*n* = 6675) and provides a more detailed exploration of the variation in CXR rates and the factors associated with this variation.

The design of this study is similar to an investigation by Mendonca *et al* that considered general practice and population characteristics with respect to urgent referrals for suspect cancer and referrals for a range of gastrointestinal endoscopic investigations.^17^ A greater degree of between-practice variation in endoscopic investigation was found to be attributable to population and practice characteristics than for CXR in the present study. In Mendonca *et al*, practice characteristics accounted for <4% of variation, whereas population characteristics accounted for proportions of variance of 17.5% to 25.1% for endoscopic investigations. In the present study, <3% of variation could be attributed to practice characteristics with population characteristics accounting for 16.4%. Of the endoscopic investigations, sigmoidoscopy is less invasive and expensive than gastroscopy and colonoscopy. CXR is less costly than all forms of gastrointestinal endoscopy and is non-invasive.^30^ CXR is also a much more common investigation.^16^^,^^31^ Investigations that are less invasive, less expensive, and more widely used are probably considered more acceptable to clinicians and patients. It is plausible that CXR is deployed more readily and for lower levels of risk of cancer than invasive investigations such as endoscopy, which might explain the lower proportion of variation in CXR rate accounted for by population factors. In the present study no association was found between GP age and CXR rate, whereas Mendonca *et al* found practices with older GPs had reduced referral rates for suspected cancers and gastroscopies.^17^

A more recent study found that urgent referrals for suspected cancer (USC) over 10 years (to 2018/2019) more than doubled to over 2 million with significant variation between practices in cancer detection.^32^ Use of urgent referral and detection of cancer was associated with larger practices and those with younger GPs, although the association with GP age became attenuated over time. In 2019/2020, of the 2.3 million urgent suspected cancer referrals only 65 000 were for suspected lung cancer (2.8% of all referrals) with 32% of lung cancers detected via USC, compared with over 50% of all cancers detected via USC.^33^

Data from the GP patient survey were explored in relation to endoscopy in Lyratzopoulos *et al*.^23^ They found that practices that scored highly for survey items relating to ease of patient access to appointments and continuity of care were correlated with reduced rates of endoscopic investigation, whereas higher investigation rates were observed in practices that had the highest scores for communication skills. Similar associations were found in the present study between patient access to appointments and CXR rate but the opposite relationship for continuity of care. Continuity of care has previously been associated with increased delay in cancer diagnosis, leading to the suggestion that ‘discontinuity’ may precipitate a fresh perspective from another clinician.^34^ The apparent disparity in the present study that demonstrated increased CXR rates in practices with high attainment for continuity of care may still be consistent with this paradigm. GPs who know their patients well may be less willing to subject their patients to invasive testing but might be more prepared to consider a less invasive test such as CXR.

### Implications for research and practice

CXR is a commonly requested investigation in primary care and is an important route to lung cancer diagnosis. As individual cancer diagnoses occur too infrequently at individual-practice level to be a reliable comparator, CXR rates may have utility as a process measure in comparing general practices’ activity pertaining to lung cancer detection and could be considered for inclusion as a cancer metric in general practice profiles.^14^^,^^15^ Although there is no consensus or guidance as to the volume of CXRs that practices should expect to undertake, individual English general practices or organisations such as Primary Care Network, may wish to access the Diagnostic Imaging Dataset and compare their annual utilisation of CXR with the median rate of 34 per 1000 patients demonstrated in this study.

This research follows previous work by reporting on associations between patient experience metrics and investigation rates.^23^ Further research may be helpful both to clarify the reasons for these associations and to determine whether patient-reported experience metrics accurately reflect objective comparisons of care between practices.

Evidence from a symptom awareness campaign during which CXR rates were increased suggested that higher volumes of imaging may contribute to a stage shift and improved survival.^13^ However, no direct ecological evidence exists to demonstrate that patients diagnosed at practices with a greater propensity to investigate with CXR benefit from earlier stage at diagnosis, as has been demonstrated for endoscopy and gastrointestinal cancer.^13^^,^^35^ Indeed, O’Dowd *et al* found no reduction in deaths within 90 days of diagnosis in practices that had higher utilisation of CXR.^28^ Further research exploring whether an association exists between practices with higher CXR rates and earlier stage at diagnosis and improved survival could be undertaken using National Cancer Registry data, as has been performed in analyses exploring practice use of urgent suspected cancer pathways and cancer outcomes.^11^^,^^12^ If such an association were demonstrated, given the low cost and high accessibility of CXR, reducing investigation thresholds for patients with symptoms in practices that currently have lower CXR rates could be a cost-effective way to improve outcomes.
